# Preliminary assessment of the utilization of durian peel liquid smoke as a natural preservative for mackerel

**DOI:** 10.12688/f1000research.18095.6

**Published:** 2019-11-08

**Authors:** Muhammad Faisal, Asri Gani, Farid Mulana

**Affiliations:** 1 Chemical Engineering Department, Syiah Kuala University, Banda Aceh, 23111, Indonesia

**Keywords:** durian peel, pyrolysis, liquid smoke, natural preservatives, TVB, organoleptic quality

## Abstract

**Background:** Durian peel is a type of biomass waste that contains cellulose, hemicelluloses, and lignin. The pyrolysis of these compounds results in production of liquid smoke which can be used as a natural preservative to replace current synthetic preservatives. This research assessed the ability of liquid smoke produced during pyrolysis of durian peel to preserve fish.

**Methods:** Dry durian peel waste underwent batch reactor pyrolysis at 340°C and 380°C, resulting in production of liquid smoke (grade 3), charcoal, and tar. This liquid smoke was then distilled at 190°C to produce grade 1 liquid smoke, which was used to preserve mackerel. The preservation process was conducted by soaking the mackerel samples in liquid smoke at 0.5, 1, 2, and 3% concentration levels followed by observations every 6 hours. Tests to determine the total volatile base nitrogen (TVB-N) content, antibacterial quality of the liquid smoke and organoleptic quality of the fish were conducted in order to assess the preservation properties of the liquid smoke.

**Results:** Tests on the antibacterial effects showed that the liquid smoke inhibited the growth of *Escherichia coli *and *Staphylococcus aureus *on fish even at low concentrations. At 54 hours, the TVB-N values remained below 30 mg nitrogen/g, indicating that the fish was still safe for human consumption. Results from the organoleptic tests showed that the concentration of liquid smoke influenced the preservation effects.

**Conclusions:** At a concentration of 2–3%, the fish samples possessed acceptable flavor, taste, color and texture for up to 48 hours of soaking. However, the best conditions were obtained with a 3% concentration of liquid smoke (produced with 340°C pyrolysis), as the fish was still considered acceptable for up to 42 hours.

## Introduction

Indonesia is located at the equator and is rich in abundant plantation produce as well as other natural resources, such as durian. Although seasonal, durian production in Indonesia continues throughout the year. High consumption of durian can lead to environmental issues because there is no proper management of durian peel waste. In general, durian peel contains a high level of cellulose (50–60%), starch (20%) and lignin ^1^, and has the potential to be used as a raw material for production of liquid smoke.

A common method to produce liquid smoke is pyrolysis, in which cellulose, lignin, and starch are processed into various chemical compounds ^2^. This process occurs in various stages: (i) hemicelluloses are disintegrated at 200–315°C, resulting in formation of furan, acetic acid and its derivatives; (ii) cellulose is disintegrated at 240–350°C, resulting in carbonic acid formation; (iii) lignin is disintegrated at 280–500°C, resulting in production of phenol, phenolic ether and its derivatives ^2^. In principle, any wood material can be used in pyrolysis to produce liquid smoke, including palm kernel shells, sugar cane fibers, empty fruit bunches, rice husks, and coconut peel ^3– 10^. Previous research ^11– 13^ has shown that the liquid smoke produced from pyrolysis of palm kernel shells contained phenol, carbonyl and other acids. These compounds have antimicrobial properties that can help preserve food ^14– 17^; they inhibit damaging and spoilage microbes and therefore increase the shelf-life of food products. In addition, liquid smoke can contribute a unique flavor, taste and color to foods. Several researchers have studied the effects of liquid smoke produced from palm kernel shells to preserve mackerel ^16^, fish ball ^18^ and tofu ^19^. However, to our knowledge, there is no research reported on the utilization of durian peel biomass as a natural preservative, despite the wide availability and potential usefulness of durian peel waste.

Fish is a staple food for people living in Indonesia’s coastal and maritime areas. The fish is usually consumed fresh or as processed products. Fish has highly nutritious and beneficial for health, however, it decomposes easily (in approximately 8 hours) due to the activity of spoilage microorganisms. Traditional fishermen use formaldehyde as a preservative to lengthen the storage life, but this compound is dangerous to human health. In recent years because of concern regarding the use of chemical preservatives, there has been much research conducted on natural antimicrobials to prevent microbial growth and food spoilage. One natural alternative is liquid smoke, which contains antimicrobial properties. The application of liquid smoke to preserve fish is simple, can be used repeatedly and has the potential to replace commonly used harmful man-made preservatives. This research aims to study the potential usefulness of liquid smoke resulting from durian peel pyrolysis as a natural mackerel preservative.

## Methods

### Liquid smoke production

Liquid smoke was produced in a pyrolysis reactor as explained in previous research ^1, 16^. As much as 3 kg of dried durian peel was placed in the reactor set at 340°C and 380°C. The resulting smoke was condensed to produce tar, charcoal and grade 3 liquid smoke. The next step was distillation at 190°C to separate the liquid smoke from tar, resulting in production of grade 1 liquid smoke. The composition of liquid smoke grade 1 can be found elsewhere ^1^. The liquid smoke grade 1 mainly contains acetic acid, phenol and small amount of other compound such as ketones, aldehydes, and carboxylic acids. While the liquid smoke grade 3 could not be used as preservative because still contain a toxic compound such as tar ^1^. The liquid smoke grade 1 was then used as the preservative by soaking mackerel in different concentrations (0.5%, 1%, 2%, and 3%) for 60 hours (untill end of preservation time). The following analysis tests were conducted during storage: measurement of TVB, organoleptic quality, antibacterial activities and total number of bacterial counts. The analyses were conducted every six hours.

### Total volatile base nitrogen (TVB-N)

TVB-N is a method to determine the freshness of fish based on its spoilage due to microbial growth and loss of fats or proteins ^20^. The TVB-N was determined according the procedure established by Susanto *et al*. with a slight modfication ^21^. TVB-N measurement was carried out by placing a Conway petri dish sideways with its lid half open inside an incubator at 35°C for 35 minutes. The petri dish contained the liquid smoke used to soak the fish, and K _2_BO _3_, and H _3_BO _3 _in each partition. After incubation, the dish was covered and shaken, before further incubation at 35°C for 8 hours. Afterwards, 0.1 ml boric acid was exposed to every indicator and left for 2 hours. Titrations with HCl (0.01 N) were performed until the color turned pink.

### Organoleptic tests

The organoleptic tests involved examining the samples using the senses of volunteer panelists, including examining the color, smell, taste and texture of the fish meat. These tests were carried out in order to identify how much people liked the liquid smoke preserved mackerel and to determine how long the fish would last. Testing was carried out in compliance with organoleptic testing standard manuals SNI 01-2346-2006 ^22^. The number of panelists used in this research was 30 people, consisting of 23 non-standard subjects (people who were not trained in performing organoleptic assessment/testing, recruited from the pool of chemical engineering students at Syiah Kuala University) and seven standard subjects (people with high sensitivity towards testing product quality, and possessed knowledge and experience in assessing product quality, recruited from the Health Laboratory, Banda Aceh). The panelists were given briefing and training prior to performing the tests. The resulting values were then processed using hedonic tests.

Average quality value: $x\;=\;\frac{\sum xi}{n}$

Where *x* = average quality scores, *x _i_* = value of organoleptic quality testing; panels *i*, and *n* = number of panelists

The statistical analysis of standard deviation was also performed on each data of the organoleptic test results for color, flavor, aroma and texture of fish during storage. For statistical analysis, One-Way ANOVA with Least Significant Different (LSD) test using the SPSS ver.22 for window was used. The authors presented the quantitative data as mean ± standard deviation (SD). Normally distributed quantitative data should be summarized as mean. Here, the SD refers to the variation in the values of the variable within the sample. The larger the SD, the greater the variability within the sample.

Flavor testing on fish was carried out after the samples had been steamed (at 90–100°C for 15 min) without changing the flavor. Table 1 describes the scale used to determine the flavor of fish samples.

**Table 1.** Organoleptic testing scale used to determine quality of fish.

Scale	Flavor	Aroma/Smell	Color	Texture
1	Very bad	Very smelly	Brown	Very soft
2	Bad	Smelly	Light brown	Soft
3	Average	Average smell	Cream colored	A little chewy
4	Good	A little smell	Light cream colored	Quite chewy
5	Very good	No smell	White	Chewy

### Antibacterial activity testing

The antibacterial activity testing was carried out to identify the activity of durian peel liquid smoke against *Escherichia coli* and *Staphylococcus aureus* bacteria. The method used was the disk diffusion (Kirby–Bauer) assay, as described by Tendencia ^23^, involving the use of Mueller Hinton (MH) media (Merck, KgaA, Germany), performed by the Health Laboratory (Banda Aceh, Indonesia). This method was chosen because of its simplicity and the ability to see the formation of inhibition zones as clear areas around the antimicrobial disks.

### Number of total bacterial counts

The total bacterial counts of mackerel was determined by Plate Count Agar (PCA) according to Indonesian standard manuals SNI 2897:2008 ^24^. The analysis was performed by the Health Laboratory (Banda Aceh, Indonesia)

## Results and discussion

### TVB-N Testing

**Figure 1.**
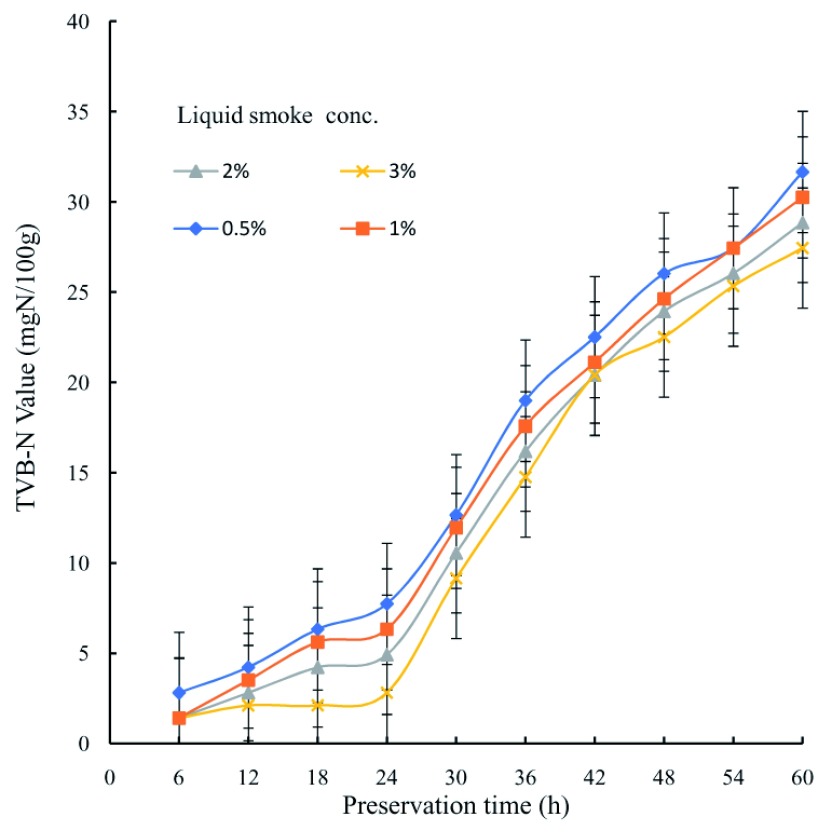
Association between preservation time, liquid smoke concentration and TVB-N value (liquid smoke was produced at 340°C).

**Figure 2.**
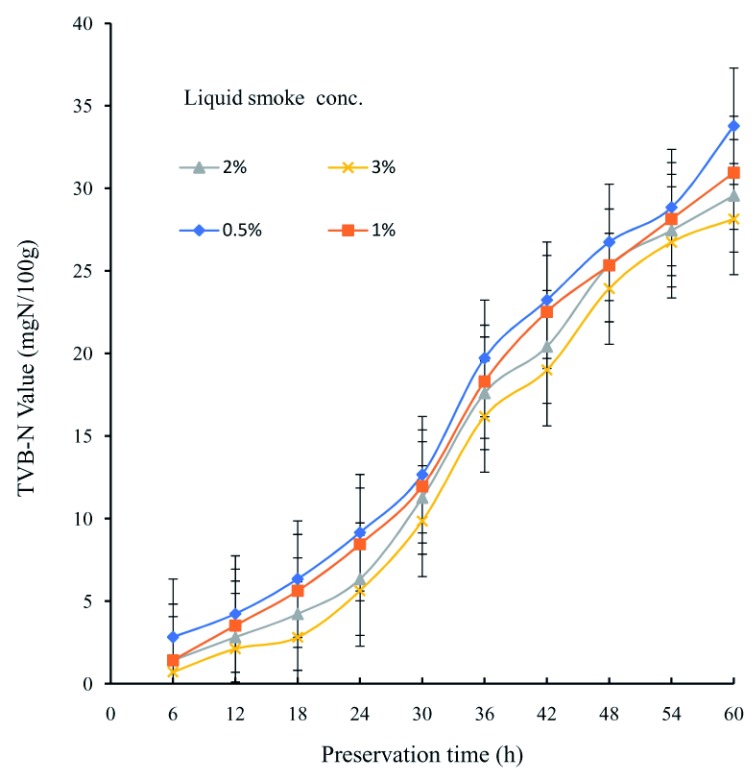
Association between preservation time, liquid smoke concentration and TVB-N value (liquid smoke was produced at 380°C).

The TVB-N values in fish after being treated with liquid smoke are shown in Figure 1 and Figure 2. The higher the concentration of liquid smoke, the lower the TVB-N value and the greater the antibacterial inhibition produced. The lowest TVB-N value was observed with 3% liquid smoke. Figure 1 shows that the TVB-N values resulting from the use of liquid smoke produced at 340°C at 0.5%, 1%, 2%, and 3% concentrations were low at 2.814, 1.407, 1.407 and 1.407 mg nitrogen/g (mgN/100g), respectively. In the meantime, liquid smoke produced at 380°C ( Figure 2) at the same concentrations resulted in the following TVB-N values: 2.814; 1.407; 1.407 and 0.7035 mgN/100 g within 6 hours. Within 54 hours, the TVB-N values for each soaking time were still within the safe limits allowed. After 60 hours, the TVB-N values increased to levels greater than 33 mgN/100g, which is above the acceptable consumer standard of 30 mgN/100g. These results are comparable to those obtained in previous research ^16^ using liquid smoke from oil palm kernel shells. A TVB-N value of 0–30 mgN/100g in fresh produce signifies good quality in compliance with National Indonesian Standards for food (SNI 01-2729-1992). TVB-N values increase due to a bacterial enzyme which degrades proteins into amino acids, and short peptide bonds resulting in production of a number of bases including, amine, ammonia, and trimethylamine which produce foul odors in foods ^25^. Longer soaking periods will result in greater bacterial activities, which in turn produce more bases and increase TVB-N values ^16^.

### Organoleptic tests

***Color.*** Results from organoleptic testing on color of samples showed that mackerel soaked in various concentrations of liquid smoke changed color depending on the soaking duration ( Table 2). With 0.5–3% liquid smoke concentrations produced at 340°C pyrolysis, the color changed from pale white to yellowish–cream in 36 hours. The same concentrations for 380°C pyrolysis caused the color to change from pale white to yellowish cream in 42 hours. A food product that has high nutritional value, good flavor and good taste will have little interest from customers if the product does not also have an attractive color. Fish soaked in 0.5–3% liquid smoke produced at 380°C had the most optimum results, probably due to a high content of phenol (1.73 wt.%) and acetic acid (8.51wt.%) compared to that of liquid smoke produced at 340°C (phenol=0.79 wt. % and acetic acid=3.40 wt. %) ^1^, which maintained the freshness of the fish ^16, 19^. For comparison, fish samples that did not receive liquid smoke treatment turned a cream color after only 8 hours. Raw data are available on Zenodo ^26^.

**Table 2.** The organoleptic test results for color of fish during storage.

Pyrolysis temperature (°C)	Liquid smoke conc. (%)	Preservation time (hours)									
		6	12	18	24	30	36	42	48	54	60
340	0.5	5 ±0.00 ^Aa^	5 ±0.00 ^Aa^	5 ±0.00 ^Aa^	5 ±0.00 ^Aa^	4.2 ±0.38 ^Aa^	3.7 ±0.48 ^Aa^	3 ±0.00 ^Aa^	2 ±0.00 ^Aa^	1.0 ±0.00 ^Aa^	1.0 ±0.00 ^Aa^
	1	5 ±0.00 ^Aa^	5 ±0.00 ^Aa^	5 ±0.00 ^Aa^	5 ±0.00 ^Aa^	4.2 ±0.38 ^Aa^	3.8 ±0.00 ^Ba^	3.4 ±0.51 ^Ba^	2.1 ±0.00 ^Ba^	1.0 ±0.38 ^Aa^	1.0 ±0.00 ^Aa^
	2	5 ±0.00 ^Aa^	5 ±0.00 ^Aa^	5 ±0.00 ^Aa^	5 ±0.00 ^Aa^	5 ±0.00 ^Ba^	4 ±0.00 ^Ca^	3.6 ±0.48 ^Ca^	2.5 ±0.48 ^Ca^	2.0 ±0.38 ^Ba^	2.0 ±0.00 ^Ba^
	3	5 ±0.00 ^Aa^	5 ±0.00 ^Aa^	5 ±0.00 ^Aa^	5 ±0.00 ^Aa^	5 ±0.00 ^Ba^	4.1 ±0.00 ^Db^	4 ±0.00 ^Da^	3.3 ±0.48 ^Da^	2.0 ±0.00 ^Ba^	2.0 ±0.00 ^Ba^
380	0.5	5 ±0.00 ^Aa^	5 ±0.00 ^Aa^	5 ±0.00 ^Aa^	5 ±0.00 ^Aa^	4.5 ±0.48 ^Bb^	4 ±0.38 ^Ab^	3.8 ±0.48 ^Bb^	2.4 ±0.38 ^Ab^	2.0 ±0.00 ^Ab^	1.0 ±0.00 ^Aa^
	1	5 ±0.00 ^Aa^	5 ±0.00 ^Aa^	5 ±0.00 ^Aa^	5 ±0.00 ^Aa^	4.2 ±0.38 ^Aa^	4 ±0.00 ^Ab^	3.8 ±0.38 ^Bb^	2.9 ±0.38 ^Bb^	2.0 ±0.00 ^Ab^	1.0 ±0.48 ^Aa^
	2	5 ±0.00 ^Aa^	5 ±0.00 ^Aa^	5 ±0.00 ^Aa^	5 ±0.00 ^Aa^	5 ±0.00 ^Ca^	4.3 ±0.48 ^Bb^	3.6 ±0.51 ^Aa^	3.2 ±0.38 ^Ca^	3.0 ±0.51 ^Bb^	2.0 ±0.38 ^Ba^
	3	5 ±0.00 ^Aa^	5 ±0.00 ^Aa^	5 ±0.00 ^Aa^	5 ±0.00 ^Aa^	5 ±0.00 ^Ca^	4 ±0.00 ^Aa^	3.9 ±0.38 ^Ca^	3.4 ±0.48 ^Da^	2.0 ±0.00 ^Aa^	2.0 ±0.38 ^Ba^

**Note:** Numbers followed by lowercase letters (vertical) with uppercase letters (horizontal) show no significant difference ( *α* <0.05). Uppercase letters: Concentration. Lowercase letters: temperature.

***Flavor and aroma.*** The hedonic test results for flavor and aroma are shown in Table 3 and Table 4. High concentrations of liquid smoke slowed down the occurrence of foul smells and bad flavors in mackerel for up to 30 hours ( Table 3 and Table 4). With regards to aroma, the use of 2–3% liquid smoke produced at 380°C maintained a desirable aroma for up to 48 hours of soaking, although the aroma grew thick (similar to the smell of liquid smoke). The smoked scent seeped into the mackerel and grew stronger due to a reduced content of acetic acid in the fish. Production of foul odors can also be used to indicate food spoilage caused by oxidation and fat oxidation lead to production of foul odors in fish ^27^. With regards to taste, liquid smoke produced a smoky smell in the fish but without smoke treatment, the deterioration in changes in taste and smell would have occurred in less than 12 hours. Saloko *et al*. ^17^ stated that the use of 5% liquid smoke in a nanocapsule from chitosan and maltodextrin had the potential to maintain the freshness of mackerel for up to 24 hours.

**Table 3.** The organoleptic test results for flavor of fish during storage.

Pyrolysis temperature (°C)	Liquid smoke conc. (%)	Preservation time (hours)									
		6	12	18	24	30	36	42	48	54	60
340	0.5	5 ±0.00 ^Aa^	5 ±0.00 ^Aa^	5 ±0.00 ^Aa^	4.4 ±0.49 ^Bb^	4.4 ±0.49 ^Bb^	3 ±0.00 ^Cb^	3 ±0.00 ^Aa^	3.1 ±0.24 ^Aa^	3 ±0.25 ^Bb^	1 ±0.49 ^Aa^
	1	5 ±0.00 ^Aa^	5 ±0.00 ^Aa^	5 ±0.00 ^Aa^	4.7 ±0.44 ^Ab^	4.5 ±0.49 ^Ab^	3 ±0.00 ^Ab^	3 ±0.00 ^Bb^	3.1 ±0.26 ^Aa^	3 ±0.49 ^Aa^	1 ±0.00 ^Aa^
	2	5 ±0.00 ^Aa^	5 ±0.00 ^Aa^	5 ±0.00 ^Aa^	4.8 ±0.41 ^Ab^	4.7 ±0.32 ^Ca^	3 ±0.00 ^Ba^	3 ±0.00 ^Da^	3.1 ±0.25 ^Ba^	3 ±0.50 ^Ca^	2 ±0.49 ^Ba^
	3	5 ±0.00 ^Aa^	5 ±0.00 ^Aa^	5 ±0.00 ^Aa^	5 ±0.00 ^Ab^	4.9 ±0.26 ^Ca^	4 ±0.00 ^Db^	3.7 ±0.45 ^Ca^	3.1 ±0.26 ^Aa^	3 ±0.44 ^Ba^	2 ±0.00 ^Aa^
380	0.5	5 ±0.00 ^Aa^	5 ±0.00 ^Aa^	5 ±0.00 ^Aa^	4.1 ±0.25 ^Aa^	4 ±0.00 ^Aa^	3 ±0.00 ^Ba^	3 ±0.00 ^Bb^	2.4 ±0.49 ^Aa^	1 ±0.00 ^Aa^	1 ±0.00 ^Aa^
	1	5 ±0.00 ^Aa^	5 ±0.00 ^Aa^	5 ±0.00 ^Aa^	4 ±0.00 ^Aa^	3.8 ±0.42 ^Aa^	3.4 ±0.49 ^Aa^	3 ±0.00 ^Aa^	3 ±0.00 ^Aa^	2 ±0.00 ^Aa^	1 ±0.00 ^Aa^
	2	5 ±0.00 ^Aa^	5 ±0.00 ^Aa^	5 ±0.00 ^Aa^	4 ±0.00 ^Aa^	4 ±0.00 ^Cb^	3.9 ±0.26 ^Db^	3.9 ±0.24 ^Db^	3.2 ±0.45 ^Ba^	3 ±0.42 ^Cb^	2 ±0.44 ^Ba^
	3	5 ±0.00 ^Aa^	5 ±0.00 ^Aa^	5 ±0.00 ^Aa^	4 ±0.00 ^Aa^	4 ±0.00 ^Ba^	4 ±0.00 ^Ca^	3.6 ±0.49 ^Cb^	3.5 ±0.49 ^Bb^	3 ±0.00 ^Ba^	2 ±0.00 ^Aa^

**Note:** Numbers followed by lowercase letters (vertical) with uppercase letters (horizontal) show no significant difference ( *α* <0.05). Uppercase letters: Concentration. Lowercase letters: temperature.

**Table 4.** The organoleptic test results for aroma of fish during storage.

Pyrolysis temperature (°C)	Liquid smoke conc. (%)	Preservation time (hours)									
		6	12	18	24	30	36	42	48	54	60
340	0.5	5 ±0.00 ^Aa^	5 ±0.00 ^Aa^	5 ±0.00 ^Aa^	4.9 ±0.23 ^Aa^	3.7 ±0.46 ^Ba^	3.4 ±0.49 ^Bb^	2.5 ±0.50 ^Aa^	2.0 ±0.00 ^Aa^	2.0 ±0.46 ^Aa^	1.0 ±0.00 ^Aa^
	1	5 ±0.00 ^Aa^	5 ±0.00 ^Aa^	5 ±0.00 ^Aa^	4.1 ±0.26 ^Bb^	3.4 ±0.50 ^Aa^	3.1 ±0.26 ^Aa^	2.7 ±0.46 ^Ba^	2.0 ±0.00 ^Aa^	1.0 ±0.00 ^Ba^	1.0 ±0.00 ^Aa^
	2	5 ±0.00 ^Aa^	5 ±0.00 ^Aa^	5 ±0.00 ^Aa^	4.1 ±0.33 ^Db^	4 ±0.00 ^Ba^	3.2 ±0.41 ^Ca^	3.1 ±0.39 ^Ca^	2.6 ±0.48 ^Ba^	2.2 ±0.41 ^Ba^	2.0 ±0.00 ^Aa^
	3	5 ±0.00 ^Aa^	5 ±0.00 ^Aa^	5 ±0.00 ^Aa^	4.1 ±0.26 ^Ca^	4 ±0.00 ^Ca^	3.9 ±0.36 ^Cb^	3.0 ±0.00 ^Cb^	2.0 ±0.00 ^Bb^	2.0 ±0.10 ^Ba^	1.0 ±0.00 ^Bb^
380	0.5	5 ±0.00 ^Aa^	5 ±0.00 ^Aa^	5 ±0.00 ^Aa^	4 ±0.00 ^Aa^	3 ±0.00 ^Aa^	3.1 ±0.26 ^Aa^	2.8 ±0.35 ^Ab^	2.0 ±0.00 ^Cb^	1.0 ±0.00 ^Ab^	1.0 ±0.00 ^Aa^
	1	5 ±0.00 ^Aa^	5 ±0.00 ^Aa^	5 ±0.00 ^Aa^	4 ±0.00 ^Aa^	3 ±0.00 ^Aa^	3 ±0.00 ^Bb^	2.0 ±0.00 ^Aa^	2.0 ±0.00 ^Aa^	1.0 ±0.00 ^Aa^	1.0 ±0.00 ^Aa^
	2	5 ±0.00 ^Aa^	5 ±0.00 ^Aa^	5 ±0.00 ^Aa^	4 ±0.00 ^Aa^	4.7 ±0.46 ^Ba^	3.8 ±0.39 ^Cb^	3.5 ±0.50 ^Ca^	3.0 ±0.00 ^Ca^	3.0 ±0.39 ^Aa^	2.0 ±0.0.5 ^Aa^
	3	5 ±0.00 ^Aa^	5 ±0.00 ^Aa^	5 ±0.00 ^Aa^	4 ±0.00 ^Aa^	4.2 ±0.42 ^Aa^	3.6 ±0.48 ^Ba^	3.2 ±0.39 ^Ba^	3.0 ±0.00 ^Ba^	2.0 ±0.00 ^Bb^	1.0 ±0.00 ^Aa^

**Note:** Numbers followed by lowercase letters (vertical) with uppercase letters (horizontal) show no significant difference ( *α* <0.05). Uppercase letters: Concentration. Lowercase letters: temperature.

***Texture.*** Texture tests can be carried out orally as well as by touching with hands aimed at feeling the texture of a food product. Table 5 shows that the best texture of mackerel occurred when 3% liquid smoke produced at 380°C was used for up to 48 hours. The fish texture was still quite chewy up to 42 hours in fish treated with 2–3% liquid smoke produced at 340°C. At both pyrolysis temperatures, the fish texture became rigid after 48 hours. At a low concentration (0.5%), the texture of the fish started to change within 36 hours. The present of acids and phenolic compounds in liquid smoke might affect the flavor, aroma, and texture of the fish ^16^. The change in texture was influenced by the speed of bacterial growth. Fish quality decreased when the texture became tender due to the effects of cathepsin and collagenase enzymes on muscle tissues. Cathepsin in fish degrades protein and causes the meat to become tender, while collagenase degrades polypeptide bonds when protein is not denatured ^27^.

**Table 5.** The organoleptic test results for texture of fish during storage.

Pyrolysis temperature (°C)	Liquid smoke conc. (%)	Preservation time (hours)									
		6	12	18	24	30	36	42	48	54	60
340	0.5	5 ±0.00 ^Aa^	5 ±0.00 ^Aa^	5 ±0.00 ^Aa^	4 ±0.00 ^Aa^	4 ±0.00 ^Ba^	3.3 ±0.42 ^Bb^	2.4 ±0.49 ^Aa^	2 ±0.00 ^Aa^	1 ±0.00 ^Aa^	1 ±0.00 ^Aa^
	1	5 ±0.00 ^Aa^	5 ±0.00 ^Aa^	5 ±0.00 ^Aa^	4.2 ±0.45 ^Bb^	3.8 ±0.41 ^Aa^	3.1 ±0.26 ^Aa^	3 ±0.00 ^Ba^	2 ±0.00 ^Aa^	2 ±0.00 ^Ba^	1 ±0.00 ^Aa^
	2	5 ±0.00 ^Aa^	5 ±0.00 ^Aa^	5 ±0.00 ^Aa^	5 ±0.00 ^Da^	4 ±0.00 ^Ba^	4 ±0.00 ^Ca^	4 ±0.00 ^Ca^	3 ±0.00 ^Ba^	2 ±0.00 ^Ba^	1 ±0.00 ^Aa^
	3	5 ±0.00 ^Aa^	5 ±0.00 ^Aa^	5 ±0.00 ^Aa^	4.7 ±0.45 ^Cb^	4.5 ±0.49 ^Ca^	4.3 ±0.54 ^Db^	4 ±0.00 ^Ca^	3 ±0.00 ^Ba^	2 ±0.00 ^Ba^	2 ±0.00 ^Bb^
380	0.5	5 ±0.00 ^Aa^	5 ±0.00 ^Aa^	5 ±0.00 ^Aa^	4 ±0.00 ^Aa^	4 ±0.00 ^Aa^	3 ±0.11 ^Aa^	3 ±0.00 ^Ab^	3 ±0.00 ^Bb^	2 ±0.00 ^Ab^	1 ±0.00 ^Aa^
	1	5 ±0.00 ^Aa^	5 ±0.00 ^Aa^	5 ±0.00 ^Aa^	4 ±0.00 ^Aa^	4 ±0.00 ^Ab^	4 ±0.12 ^Bb^	3 ±0.00 ^Aa^	2 ±0.00 ^Aa^	2 ±0.00 ^Ab^	1 ±0.00 ^Aa^
	2	5 ±0.00 ^Aa^	5 ±0.00 ^Aa^	5 ±0.00 ^Aa^	4.9 ±0.26 ^Ba^	4.4 ±0.49 ^Bb^	4.2 ±0.42 ^Cb^	4 ±0.00 ^Ca^	3 ±0.00 ^Aa^	2 ±0.00 ^Aa^	1 ±0.00 ^Aa^
	3	5 ±0.00 ^Aa^	5 ±0.00 ^Aa^	5 ±0.00 ^Aa^	4 ±0.00 ^Aa^	4 ±0.00 ^Aa^	4 ±0.00 ^Ba^	3.7 ±0.44 ^Ba^	3.6 ±0.32 ^Cb^	3 ±0.00 ^Ba^	1 ±0.00 ^Aa^

**Note:** Numbers followed by lowercase letters (vertical) with uppercase letters (horizontal) show no significant difference ( *α* <0.05). Uppercase letters: Concentration. Lowercase letters: temperature.

The results of SD calculation showed that a constant reproducibility SD was only obtained at 18 (eighteen) hours of preservation time on the organoleptic test results for color, flavor, aroma and texture of fish during storage. For preservation time from 24 hours until 60 hours, the standard deviation of the organoleptic test results for color, flavor, aroma and texture had fewer and more scattered data ranging of 0.00 to 0.50. These small standard deviation data indicated that generally the all-organoleptic test results are acceptable.

### Anti-bacterial activity testing

Table 6 shows the effects of liquid smoke on bacterial growth ( *E. coli* and *S. aureus*). A concentration of 0.5% liquid smoke produced at 340°C did not inhibit bacterial growth; however, bacterial inhibiting properties were shown at a concentration of 1%. The same antibacterial properties were seen in liquid smoke produced at 380°C even at lower concentrations. Kim *et al*. ^28^ stated that the use of 0.1–1% liquid smoke produced from rice husks inhibited *Salmonella* growth while Milly *et al*. ^29^ demonstrated that 1.5–9% liquid smoke produced from cinnamon can also inhibit bacteria. In addition, Saloko *et al*. ^17^ showed that 5% liquid smoke from chitosan and maltodextrin in a nanocapsule inhibited *E. coli* and *P. fluorescens* growth *.* The type of wood biomass used to produce liquid smoke will result in production of different phenol, carbonyl and acid compounds, which in turn will determine the antibacterial properties, as well as the sensitivity of the pathogenic bacteria to the liquid smoke raw material ^14^.

**Table 6.** Antibacterial effects ( *E. coli and S. aureus*) of different concentrations of liquid smoke.

Pyrolysis temperature (°C)	Bacterial activity			
	0.5% liquid smoke	1% liquid smoke	2% liquid smoke	3% liquid smoke
340	Non-inhibitor	Inhibitor	Inhibitor	Inhibitor
380	Inhibitor	Inhibitor	Inhibitor	Inhibitor

### Number of total bacterial counts

The total number of bacterial counts in the mackerel will determine whether or not the product is acceptable for human consumption. Plate count agar (PCA) was used to determine the total bacterial counts on mackerel samples. The number of microbes present after soaking must fall within the safe limits for consumption, namely 5×10 ^5^ colonies/g, in accordance with SNI 02-2725-1992 ^30^. Table 7 shows that 12 hours of soaking did not result in any significant changes in the number of counts; levels ranged from 3.2×10 ^5^ to 3.5×10 ^5 ^colonies/g, indicating that the products were considered to be safe. However, after 42 hours the number of colonies increased to levels of 5.02 × 10 ^5 ^ colonies/g, making the fish unsafe for human consumption. Liquid smoke can inhibit the growth of bacteria due to the presence of phenols, acids and carbonyl compounds working together to inhibit degradation and spoilage. In particular, acetic acid can penetrate the cell membrane and neutralize the pH gradient ^31^.

**Table 7.** Effects of different concentrations of liquid smoke on total bacterial counts on fish.

Time (hours)	Pyrolysis temperature (°C)	Number of colonies (×10 ^5^colony/g)			
		0.5% liquid smoke	1% liquid smoke	2% liquid smoke	3% liquid smoke
12	340	3.48	3.3	3.25	3.15
	380	3.5	3.46	3.37	3.26
18	340	3.44	3.37	3.33	3.10
	380	3.82	3.75	3.69	3.50
24	340	4.44	4.2	4.08	3.92
	380	4.92	4.60	4.36	4.04
36	340	4.64	4.58	4.54	4.42
	380	4.92	4.72	4.44	4.68
42	340	4.98	4.84	4.64	4.58
	380	5.02	5.26	5.12	4.96

## Conclusion

Smoke produced from durian peel pyrolysis had inhibitory effects against bacteria even when applied at low concentrations. This showed that the liquid smoke treatment had the potential to be used as a food preservative, especially for fish. The organoleptic tests carried out showed that the preservation of mackerel depended on the concentration and pyrolysis temperature used during the liquid smoke production. At a concentration of 3% liquid smoke produced at 340°C, the fish stayed acceptable for consumption for 42 hours, based on its color (cream), flavor, aroma and texture, and the total bacterial counts were also acceptable and within the safe limits. Meanwhile, TVB-N tests showed that the fish remained of acceptable quality for 54 hours, with a TVB-N value of less than 30 mgN/100g.

## Data availability

Data associated with this study, stratified by table, are available on Zenodo. Data include raw organoleptic test results for the quality of fish and effects of liquid smoke on bacterial counts. Bacterial count data are provided as mean values, since this is the output generated by the external Health Laboratory. DOI: https://doi.org/10.5281/zenodo.2556482 ^26^.

Data are available under the terms of the Creative Commons Attribution 4.0 International license (CC-BY 4.0).
