# Supplementary material for: Preliminary assessment of the utilization of durian peel liquid smoke as a natural preservative for mackerel
Source: F1000Res. 2019 Nov 8;8:240. Originally published 2019 Mar 1. [Version 6] doi: 10.12688/f1000research.18095.6 (PMC6961411; doi:10.12688/f1000research.18095.6)
Supplement: Peer review history

# Peer review: Reviewer response for version 6

**DOI:** 10.5256/f1000research.23383.r56387

**Published:** 2020-01-13

**Authors:** Maizirwan Mel

**Affiliations:** 1 Department of Biotechnology Engineering, Faculty of Engineering, International Islamic University of Malaysia, Kuala Lumpur, Malaysia

The paper is good presented and used enough references to support the discussion.

 The experiment done is not used in the complete design of the experiment (DOE) but some of the results use statistical analysis. They used OFAT design.

 The methodology has discussed well and properly such as on the preparation of durian peel (raw material),  the process of pyrolysis, analysis of smoke (product), antibacterial testing.

 It is a good paper and can be applied in the agriculture industry sector.

I confirm that I have read this submission and believe that I have an appropriate level of expertise to confirm that it is of an acceptable scientific standard.

# Peer review: Reviewer response for version 6

**DOI:** 10.5256/f1000research.23383.r56345

**Published:** 2019-11-12

**Authors:** Maryam Mahmoudzadeh

**Affiliations:** 1 Nutrition Research Center and Department of Food Science and Technology, Faculty of Nutrition and Food Science, Tabriz University of Medical Sciences, Tabriz, Iran

The manuscript is in acceptable level for indexing.

I confirm that I have read this submission and believe that I have an appropriate level of expertise to confirm that it is of an acceptable scientific standard.

# Peer review: Reviewer response for version 5

**DOI:** 10.5256/f1000research.23146.r55373

**Published:** 2019-10-29

**Authors:** Maryam Mahmoudzadeh

**Affiliations:** 1 Nutrition Research Center and Department of Food Science and Technology, Faculty of Nutrition and Food Science, Tabriz University of Medical Sciences, Tabriz, Iran

The manuscript entitled "Preliminary assessment of the utilization of durian peel liquid smoke as a natural preservative for mackerel" is in standard form now, but the authors need to do these two corrections:

1. Mention as subscript below each table what the uppercase and lowercase letters show.
2. Change the scale of figures especially in y-axial so that the error bar of each treatment is clearly identified.

I confirm that I have read this submission and believe that I have an appropriate level of expertise to confirm that it is of an acceptable scientific standard, however I have significant reservations, as outlined above.

# Peer review: Reviewer response for version 4

**DOI:** 10.5256/f1000research.22511.r53558

**Published:** 2019-09-18

**Authors:** Maryam Mahmoudzadeh

**Affiliations:** 1 Nutrition Research Center and Department of Food Science and Technology, Faculty of Nutrition and Food Science, Tabriz University of Medical Sciences, Tabriz, Iran

Unfortunately the issues are not yet resolved:

1. You need to add lowercase letters for your table data.
2. Your figures should display the Standard Errors.

I confirm that I have read this submission and believe that I have an appropriate level of expertise to confirm that it is of an acceptable scientific standard, however I have significant reservations, as outlined above.

# Response

**Published:** 2019-10-16

**Authors:** Muhammad Faisal

**Affiliations:** Syiah Kuala University, Indonesia

1. Lowercase letters have been added to Table 2-5.

2. The Figures have been revised with addition of bar errors.

# Peer review: Reviewer response for version 3

**DOI:** 10.5256/f1000research.22331.r52760

**Published:** 2019-08-27

**Authors:** Maryam Mahmoudzadeh

**Affiliations:** 1 Nutrition Research Center and Department of Food Science and Technology, Faculty of Nutrition and Food Science, Tabriz University of Medical Sciences, Tabriz, Iran

Please:

- Add your statistical method and also used software to method and material section.
- Statistical comparisons should be shown by lowercase letters on tables data.

I confirm that I have read this submission and believe that I have an appropriate level of expertise to confirm that it is of an acceptable scientific standard, however I have significant reservations, as outlined above.

# Response

**Published:** 2019-08-31

**Authors:** Muhammad Faisal

**Affiliations:** Syiah Kuala University, Indonesia

1. Standard deviation was calculated by using Microsoft Excel for Windows version 14.0.   

       2. The Tables 2-5 have been revised

# Peer review: Reviewer response for version 2

**DOI:** 10.5256/f1000research.21959.r51449

**Published:** 2019-07-30

**Authors:** Maryam Mahmoudzadeh

**Affiliations:** 1 Nutrition Research Center and Department of Food Science and Technology, Faculty of Nutrition and Food Science, Tabriz University of Medical Sciences, Tabriz, Iran

- It is recommended to add summarized details of your previous work such as general composition differences between 2 smokes and then compare observed effects with compositional differences.
- About comment 7: mention your explanation obviously in the text.
- Finally: I don't see any standard deviation in your given data, and without statistical comparison we couldn't have any claim. You should add SD to data and compare them statistically; this is very important in a scientific article.

I confirm that I have read this submission and believe that I have an appropriate level of expertise to confirm that it is of an acceptable scientific standard, however I have significant reservations, as outlined above.

# Response

**Published:** 2019-08-14

**Authors:** Muhammad Faisal

**Affiliations:** Syiah Kuala University, Indonesia

1. The summary of differences between 2 liquid smokes have been added.

The liquid smoke grade 1 mainly contains acetic acid (3.4 - 8.5 wt.%) and phenol (0.79 -1.73 wt.%). The liquid smoke grade 3 could not be used as preservative because it still contains a toxic compound such as tar (added in experimental section). The pyrolysis temperature affected the composition of liquid smoke. The observed effect was because of the presence of phenol and acetic acid in the liquid smoke, and has already been inserted in the discussion.

2. Thank you very much, we have mentioned clearly in the manuscript.

We used the soaking method for preservation. Therefore, the liquid smoke grade 1 was used as the preservative by soaking mackerel in different concentrations (0.5%, 1%, 2%, and 3%) for 60 hours (until end of preservation time). The analyses were conducted every six hours.

3. We have revised the Table and added SD data in the manuscript. Discussions have been inserted. Thank you very much.

# Peer review: Reviewer response for version 1

**DOI:** 10.5256/f1000research.19788.r50707

**Published:** 2019-07-15

**Authors:** Maryam Mahmoudzadeh

**Affiliations:** 1 Nutrition Research Center and Department of Food Science and Technology, Faculty of Nutrition and Food Science, Tabriz University of Medical Sciences, Tabriz, Iran

1. What was your grade 1 liquid smoke composition? I think the biggest gap of your work is the lack of any characterization method for liquid smoke.
2. According to which reference you have chosen the TVBN method?
3. As far as I know, "TVBN" is correct, not "TVB".
4. What is the compositional difference between 340°C and 380°C smoke?
5. Figure numbers are inaccurately typed in the text - where is Figure 3?
6. TVBN data reported in the text are equal for 2 temperatures.
7. How long did you soak treatments in liquid smoke?
8. Where is the total count assay in the method and materials section?
9. You should have control samples in all exams, I couldn't see anything like this.
10. If you have done the antibacterial test by discs, why didn't you explain it in method and materials? Also, the reported results in Table 6 are not acceptable (you should report inhibition percent data).
11. There isn’t any statistical comparison in your work, and I think a big mistake like this is not acceptable.

I confirm that I have read this submission and believe that I have an appropriate level of expertise to state that I do not consider it to be of an acceptable scientific standard, for reasons outlined above.

# Response

**Published:** 2019-07-17

**Authors:** Muhammad Faisal

**Affiliations:** Syiah Kuala University, Indonesia

1. The composition of liquid smoke grade 1 produced at various temperatures has been published in our previous work. Please see reference 1. The composition includes; 2-Propanone, Acetic acid, 2,4-Pentadienenitrile, 2-Oxepanone, Phenolic compound etc. We have added the reference information in the manuscripts.
2. The TVB-N was determined according to the procedure established by Susanto *et al. *with a slight modification. We have added the reference in the manuscript.
3. Thank you very much for your correction, the TVB has been changed to TVB-N.
4. The composition of liquid smoke at various temperature pyrolysis has been discussed in a previous article (see ref. 1). Analyses results showed that the temperature pyrolysis affected the chemical composition of liquid smoke. For example; at 340°C, liquid smoke contains Phenol=0.79 % and Acetic Acid=3.4%, at 380°C, liquid smoke contains Phenol=1.73 % and Acetic Acid=8.51%.
5. Thank you very much for your correction. We have revised the Figure 3 to become Figure 2.
6. The data is correct, because these data are at 6 hours. The TVB-N values are still low for all liquid smoke. The TVB-N then increased with increasing preservation time.
7. We used soaking method for preservation, so the fish were soaked until end of preservation time.
8. We have added the information of total bacterial counts in the method.
9. Without preservation with liquid smoke, the fish can stand only to about 8 hours. This information can be found in the manuscript. I hope this information can be used as a control comparison. We only focused on after treatment.
10. We are very sorry about this matter. We only conducted simple analysis for the antibacterial test. Detailed analysis will be done in the future.
11. We are very sorry for comparison with other researches, because this investigation is still in a preliminary step. And to our knowledge the application of durian peel liquid smoke for fish preservation is not available in the literature. Therefore we only compare our results from a few related references.
